# Bilateral transfer of motor performance as a function of motor imagery training: a systematic review and meta-analysis

**DOI:** 10.3389/fpsyg.2023.1187175

**Published:** 2023-06-02

**Authors:** Wan X. Yao, Sha Ge, John Q. Zhang, Parisa Hemmat, Bo Y. Jiang, Xiao J. Liu, Xing Lu, Zayd Yaghi, Guang H. Yue

**Affiliations:** ^1^Department of Kinesiology, College for Health, Community, and Policy, The University of Texas at San Antonio, San Antonio, TX, United States; ^2^College of Sports Science, Tianjin Normal University, Tianjin, China; ^3^School of Public Health, Jilin Medical University, Jilin, China; ^4^College of Art, Beijing Sport University, Beijing, China; ^5^Center for Mobility and Rehabilitation Engineering Research, Kessler Foundation, West Orange, NJ, United States; ^6^Rutgers New Jersey Medical School, Rutgers, The State University of New Jersey, Newark, NJ, United States

**Keywords:** motor imagery training, mental practice, bilateral transfer, interlimb transfer, cross education, motor learning, muscle strength, motor performance

## Abstract

**Objective:**

The objective of this review was to evaluate the efficacy of mental imagery training (MIT) in promoting bilateral transfer (BT) of motor performance for healthy subjects.

**Data sources:**

We searched 6 online-databases (Jul-Dec 2022) using terms: “mental practice,” “motor imagery training,” “motor imagery practice,” “mental training,” “movement imagery,” “cognitive training,” “bilateral transfer,” “interlimb transfer,” “cross education,” “motor learning,” “strength,” “force” and “motor performance.”

**Study selection and data extraction:**

We selected randomized-controlled studies that examined the effect of MIT on BT. Two reviewers independently determined if each study met the inclusion criteria for the review. Disagreements were resolved through discussion and, if necessary, by a third reviewer. A total of 9 articles out of 728 initially identified studies were chosen for the meta-analysis.

**Data synthesis:**

The meta-analysis included 14 studies for the comparison between MIT and no-exercise control (CTR) and 15 studies for the comparison between MIT and physical training (PT).

**Results:**

MIT showed significant benefit in inducing BT compared to CTR (ES = 0.78, 95% CI = 0.57–0.98). The effect of MIT on BT was similar to that of PT (ES = –0.02, 95% CI = –0.15–0.17). Subgroup analyses showed that internal MIT (IMIT) was more effective (ES = 2.17, 95% CI = 1.57–2.76) than external MIT (EMIT) (ES = 0.95, 95% CI = 0.74–1.17), and mixed-task (ES = 1.68, 95% CI = 1.26–2.11) was more effective than mirror-task (ES = 0.46, 95% CI = 0.14–0.78) and normal-task (ES = 0.56, 95% CI = 0.23–0.90). No significant difference was found between transfer from dominant limb (DL) to non-dominant limb (NDL) (ES = 0.67, 95% CI = 0.37–0.97) and NDL to DL (ES = 0.87, 95% CI = 0.59–1.15).

**Conclusion:**

This review concludes that MIT can serve as a valuable alternative or supplement to PT in facilitating BT effects. Notably, IMIT is preferable to EMIT, and interventions incorporating tasks that have access to both intrinsic and extrinsic coordinates (mixed-task) are preferred over those that involve only one of the two coordinates (mirror-task or normal-task). These findings have implications for rehabilitation of patients such as stroke survivors.

## Introduction

Bilateral Transfer (BT), also known as cross-education or intermanual transfer, refers to the transfer of the improved performance of motor activities from a trained limb to the contralateral untrained limb (Zhou, 2000; Farthing, 2009). This phenomenon has been studied for over a century, with Volkmann’s study in 1858 being one of the earliest recorded investigations (Volkmann, 1858). Since then, many studies have investigated the efficacy of BT in improving the performance of learned motor tasks with untrained limbs (Scripture et al., 1894; Cook, 1933) and examined the neuromechanisms that underlie it (Imamizu and Shimojo, 1995; Carroll et al., 2006; Lee et al., 2010; Ruddy et al., 2017; Calvert and Carson, 2022). Moreover, the field has extensively investigated the characteristics of transfer-direction, which involves transferring from the dominant limb (DL) to the nondominant limb (NDL) or from NDL to DL (Wang and Sainburg, 2006; Sainburg et al., 2016), as well as transfer-type, which includes intrinsic-coordinates transfer and extrinsic-coordinates transfer (Lange et al., 2004, 2006).

It has been proposed that motor learning results in two coordinates: extrinsic and intrinsic (Kawato et al., 1988; Andersen et al., 1993). Acquaintance of the two coordinates occurs in the early stage of learning and is refined in the later stages through appropriate training programs (Kawato et al., 1988; Andersen et al., 1993; Battaglia et al., 2020). Extrinsic coordinates relate to visual–spatial contents such as the location of an object in the subject’s immediate surroundings, while the intrinsic coordinates describe the dynamical relationships of body segments. In a BT scenario, the extrinsic coordinates are similar when executing a learned task with the untrained limb in its original orientation (normal-task) as with the trained limb. However, performing the task with the untrained limb in a mirror orientation (mirror-task) results in intrinsic coordinates similar to those used in the learned task with the trained limb (Lange et al., 2004). Hence, the precise execution of transferred movements from the trained to the untrained limb relies on information encoded in both extrinsic and intrinsic coordinates. Likewise, in mirror-task, it is essential to retrieve intrinsic coordinates and adjust extrinsic coordinates for successful execution (Lange et al., 2004, 2006). An intriguing observation was made when examining right-handed individuals who underwent physical training (PT) as an exercise protocol. In such cases, the untrained left limb (NDL) benefits almost equally from the trained right limb (DL) in both normal- and mirror-transfer tasks. However, a benefit for the untrained DL from the trained NDL is mostly observed in normal-tasks (Parlow and Kinsbourne, 1990; Lange et al., 2004, 2006).

In addition to the mirror- and normal-tasks, a new type of transfer task, called the mixed-task, should be considered. This task involves recalling both intrinsic and extrinsic coordinates. For instance, following training the right upper limb with elbow flexion movement, both extrinsic and intrinsic coordinates can be accessed and utilized later by the untrained left upper limb performing the same elbow flexion movement. To date, no studies have compared the transfer effect between mixed-task and mirror-task or mixed-task and normal-task. Understanding the impact of different types of tasks on the transfer would enable practitioners, such as therapists and coaches, to design practice strategies that optimize transfer performance of the untrained limb.

While most studies have used overt PT, a few have tested the effectiveness of covert PT, such as motor imagery training (MIT) in inducing BT in motor skill learning (Taktek et al., 2008; Amemiya et al., 2010; Lohse et al., 2010; Asa et al., 2014; Land et al., 2016; Dahm et al., 2022) and muscle strength enhancement (Yue and Cole, 1992; Farthing, 2009; Alenezi, 2018; Bouguetoch et al., 2021). It should be noted that understanding the role of MIT in inducing BT is an important area of research that has both theoretical and practical implications (Magill and Anderson, 2021). It has the potential to enhance our understanding of how the brain controls movement and to provide new rehabilitation strategies for individuals such as stroke survivors and older adults with difficulties to participate in traditional physical training. Although outside the scope of the current study, it is worth noting that there are other strategies beyond bilateral transfer that have been explored for treating patients such as stroke survivors. Mirror therapy (Thieme et al., 2018; Gandhi et al., 2020) and prismatic adaptations (Shiraishi et al., 2008; Mizuno et al., 2011; Bonaventura et al., 2020) are examples of such strategies.

MIT involves repetitively applying motor imagery of motor skill performance or strong muscle contractions. Overall, MIT has been shown to be better than a no-exercise control group but less effective than PT in improving motor skill performance (Feltz and Landers, 1983; Driskell et al., 1994; Toth et al., 2020) and muscle force (Paravlic et al., 2018; Liu et al., 2023). Neural adaptations induced by the MIT are the likely explanation for the beneficial effects because no apparent physical activities take place in MIT (Yue and Cole, 1992; Yao et al., 2013). Furthermore, a review by Toth et al. (2020) shows that internal MIT (IMIT), also known as kinesthetic or first-person imagery training, is more effective than external MIT (EMIT) in motor skill learning. IMIT requires individuals to repetitively imagine performing the exercise from within their body, while EMIT requires individuals to repetitively imagine performing the task from outside their body (i.e., from a third-person perspective, in which the person (the external imager) watches motor performance by another individual). The study by Ranganathan et al. (2004) demonstrates that performing internal motor imagery elicits significantly higher physiological responses such as heart rate and respiration rate, in comparison to external motor imagery. Additionally, Yao et al. (2013) found that IMIT leads to a significant elevation in movement-related cortical potentials in motor-related cortical areas such as M1 and supplementary cortices after 6 weeks of training, which was not observed with EMIT.

As aforementioned, several studies have attempted to determine the effectiveness of MIT on inducing BT. However, due to relatively small sample sizes and inconsistent findings, it has been challenging to draw a definitive conclusion about the overall effect of MIT on BT. A systematic review and meta-analysis would help shed light on the overall effectiveness of MIT on inducing BT effect, and consequently provide robust scientific evidence for practitioners such as coaches and physical therapists to design effective exercise and treatment plans for improving motor performance. To our knowledge, no other meta-analysis has been conducted on the topic. Therefore, this study aimed to fill this gap in the literature by conducting a systematic review and meta-analysis of randomized-controlled trials to investigate the potential impacts of MIT on BT of motor performance. Specifically, the research question was: Does evidence from randomized-controlled trials show that MIT leads to different impact on BT compared to no-exercise and PT? In addition, the study investigated the impact of MIT on several moderators, including transfer-direction (DL ➔ NDL vs. NDL ➔ DL), transfer-type (Mirror vs. Normal tasks vs. Mixed tasks), and MIT-type (IMIT vs. EMIT). The hypothesis of this study was that MIT would have a beneficial impact on BT based on previous findings that PT could induce neural adaptations that resulted in positive BT (Carroll et al., 2006, 2008; Farthing, 2009; Lee et al., 2010; Carroll et al., 2011; Zult et al., 2014), and MIT led to similar neural adaptations as PT (Hinshaw, 1991; Yue and Cole, 1992; Decety, 1996; Decety and Grezes, 1999; Ranganathan et al., 2004; Hétu et al., 2013; Yao et al., 2013). Thus, it was reasonable to expect that the neural adaptations obtained from MIT on the trained limb could transfer to the untrained limb and result in positive BT.

## Research methods

This review adhered to the systematic review checklists and guidelines outlined in the PRISMA Statement 2020 (Page et al., 2021). The procedures used in this review were similar to those described in the study by Liu et al. (2023).

### Eligibility criteria

The study used the PICOS (population, intervention, comparison, outcome, and study design) framework to establish eligibility criteria (Amir-Behghadami and Janati, 2020).

#### Inclusion criteria

1) Population: Individuals of any gender and age who were in good health, 2) Intervention: The MIT could be administered along or in combination with PT. The studies chosen for inclusion in the review must have at least one no-exercise CTR and/or one group receiving PT, 3) Comparison: The study compared motor performance between a) the intervention type (i.e., MIT vs. CTR, and MIT vs. PT), b) transferred limb (dominant limb (DL) to nondominant limb (NDL) vs. NDL to DL), c) transfer type (mirror vs. normal vs. mixed), and d) MIT type (IMIT vs. EMIT), 4) Outcome: The study must have either the post-innervation values (PIV) or the change-from-baseline values (CBV) of BT data. CBV was prioritized for further analysis if both PIV and CBV were available (see Higgins et al., 2022 for the rationals behind the decision, Deeks et al., 2022), and 5) Study design: a) The study must be a randomized controlled trial; b) To be eligible for inclusion, the study must have been published in a peer-reviewed journal or be an unpublished dissertation prior to December 30, 2022; and c) The study must have tested the efficacy of the effect of MIT on BT.

#### Exclusion criteria

1) articles published in languages other than English, 2) non-randomized studies or studies involving participants with healthy issues, and 3) studies lacking sufficient information to calculate effect sizes, such as missing means and standard deviations.

### Search sources, screening strategy, and selection process

A computerized search of multiple databases, including PubMed/Medline, ERIC, Web of Science, Google Scholar, ScienceDirect, and ProQuest, was conducted by two researchers (WXY and SG). The search was performed using Boolean operators, such as “AND,” “OR,” and “NOT,” along with a range of relevant keywords, including “motor imagery training,” “motor imagery practice,” “mental practice,” “mental training,” “movement imagery,” “cognitive training,” “bilateral transfer,” “interlimb transfer,” “cross education,” “motor learning,” “strength,” “force,” and “motor performance.” Additionally, the authors manually searched reference lists of obtained articles (reference and author tracking) for further relevant studies. The search was concluded on December 30, 2022.

**Figure 1:**
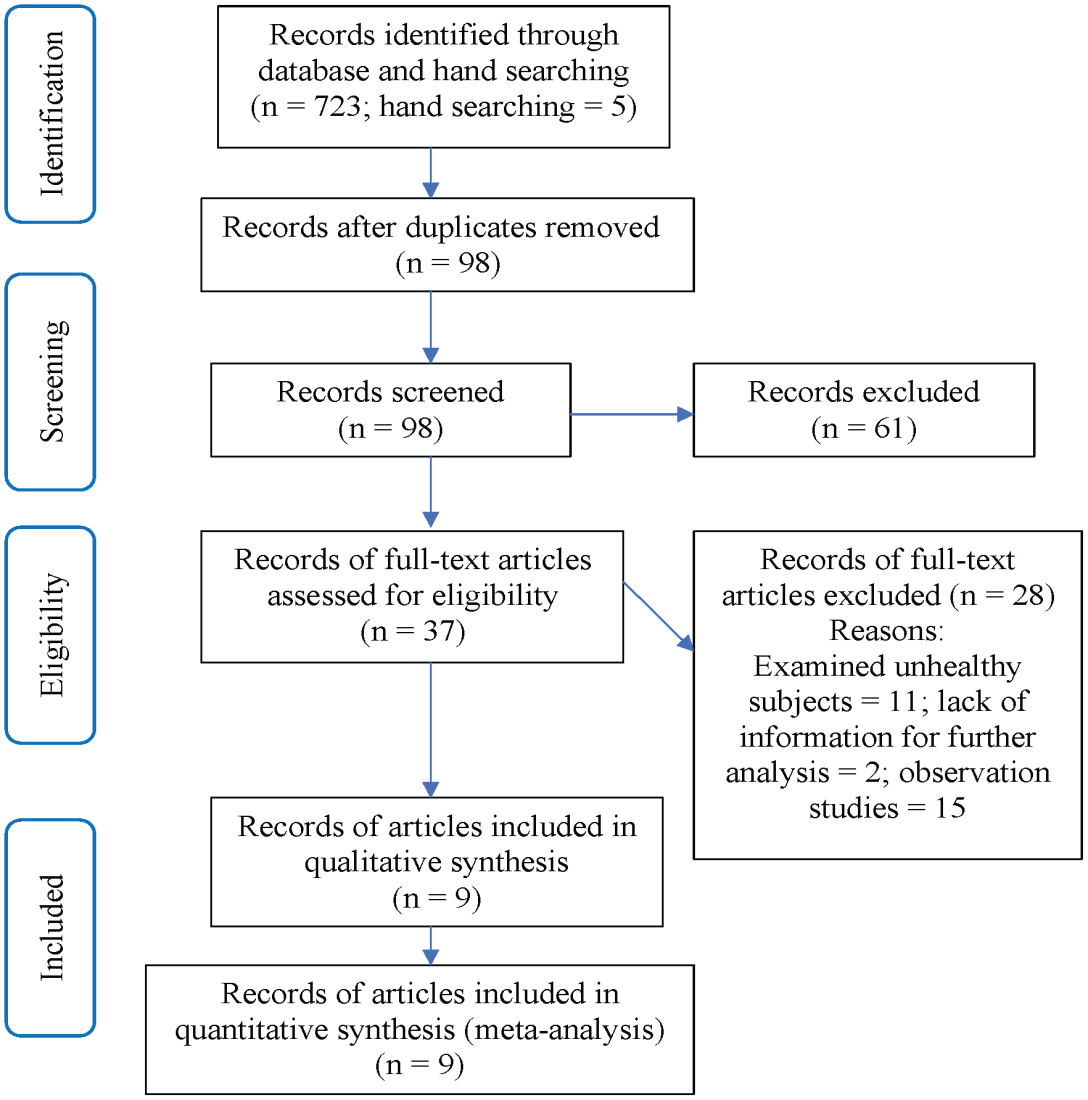
Study selection process.

The search process followed the steps outlined below: first, the two reviewers (WXY and SG) analyzed the titles and abstracts based on the predetermined inclusion and exclusion criteria. Then, the full texts of the papers that met the inclusion criteria were retrieved and carefully reviewed by the two reviewers to decide which articles to include in the meta-analysis. In cases where the suitability of a study was uncertain, the two reviewers re-examined it and reached a decision through discussion. If they could not reach a consensus, a third reviewer (GHY) was consulted to make the final decision. Please see Figure 1 for a clearer representation of the selection process.

### Data extraction

One reviewer (WXY) extracted the data into a spreadsheet developed based on the Template for Intervention Description and Replication checklist (Hoffmann et al., 2014), which was then checked by a second reviewer (SG). The data extracted from the selected studies included the gender of the subjects, the sample size in each group, the tasks used, the type of transfer assessed, the direction of transfer assessed, the frequency of MIT sessions, the total trials and total time of the entire MIT intervention, and the major findings reported in each study. In cases where the manuscript did not present the necessary data, the authors were contacted for the information. For studies where data were presented in figures, the WebPlotDigitizer software (version 4.5, August 2021; Ankit Rohatgi; Pacifica, California, USA) was utilized to extract the data.

### Risk of bias (methodological quality) assessment

Two reviewers (WXY and SG) independently evaluated the quality of the included studies using the PEDro scores. The PEDro scale evaluates the quality of randomized controlled trials based on 11 items. The total score ranges from 0 to 10 (the question of whether the study is a randomized-controlled trial is not included in the scoring), with higher scores indicating better methodological quality. The studies were categorized into three groups based on the PEDro score: poor (<4), fair (4–6), or good (7–10) quality trials (Maher et al., 2003). Any discrepancies between the two reviewers were first resolved through discussion. If agreements could not be reached, a third reviewer (GHY) would arbitrate.

### Reporting bias

A funnel plot was applied to visualize any evidence of publication bias, and if present, Egger’s regression test was conducted to provide statistical evidence. The authors also provided their opinions on the potential sources of asymmetry (Page et al., 2022).

### Synthesis and effect measures

The inclusion and exclusion criteria were predetermined, and studies were selected based on these criteria. The selected studies’ outcomes were grouped according to their innervation types: MIT, PT, and CTR. R-Studio (version 1.4.1717–3, “Juliet Rose” for macOS), a statistical computing language, was used to perform the meta-analyses. The primary packages used in the study were “meta,” “rmeta,” and “metafor.” One reviewer (WXY) undertook the meta-analysis of various continuous measures of motor performance, presenting results as point estimates and 95% confidence intervals (CI), using SMD (standardized mean differences). Heterogeneity was evaluated through visual inspection of forest plots and the calculation of the chi-square and I^2^ statistics. Subgroup analyses were performed to determine the similarities and/or differences in MIT effect on BT between the two transfer-direction groups (i.e., DL ➔NDL vs. NDL ➔DL), the two MIT-type groups (IMIT vs. EMIT), and three transfer-type groups (i.e., mirror vs. normal vs. mixed). Significance of the subgroup differences was determined by calculating Q-tests (Harrer et al., 2022).

## Results

### Study selection

The online database and manual literature search identified a total of 728 articles (Figure 1). After removing duplicates and screening articles based on their titles and abstracts, 37 articles remained. The two reviewers (WXY and SG) conducted an independent evaluation of these articles. Ultimately, we included 9 articles that reported on 29 studies (14 for MIT vs. Control and 15 for MIT vs. PT) in the systematic review and meta-analysis.

### Risk of bias (methodological quality) assessment

Table 1 displays the PEDro scores for all included studies. Overall, the studies included in the analysis were deemed to be of high quality, with PEDro scores of 7.00. Specifically, all studies scored points for meeting the following criteria: eligibility criteria, similarity of groups at baseline, blinding of subjects, measures of at least one key outcome were obtained from more than 85% of the subjects, intention-to-treat analysis performed, statistical comparisons of between-group reported for at least one key outcome, and measures of variability reported for at least one key outcome. However, none of the included studies satisfied the following criteria: concealed allocation and blinded assessors/experimenters.

**Table 1:** Characteristics and major outcomes of the included studies.

Articles	Pedro Score	Gender Age: Mean ± SD	Sample size	Tasks	TFR Type	TFR DIR	WKs of MIT	SESS Per WK	Total Trials	Total Time (min)	Key findings (%)
Alenezi (2018)	7	Male & Female 25.5 ± 3.99	MIT = 15 PT = 15	IMIT with hip abduction; isometric	Mixed	DH ➔ NDH	2	5	350	~150	MIT 6.92 PT NC
Amemiya et al. (2010)	7	Male & Female 21.4 ± NR	MIT = 11 PT = 11 CTR = 11	IMIT with sequential tapping task	Mirror	NDH ➔ DH	1	1	NR	~5	MIT 105 CTR 53
Asa et al. (2014)	7	Male & Female 9.9 ± 0.2	MIT = 12 PT = 12 CTR = 12	Finger-to-thumb opposition sequence task	Mixed	DH ➔ NDH	1	1	120	~30	MIT 65 PT 42 CTR 13
Bouguetoch et al., 2021	7	Male & Female 19–36	MIT = 9 CTR = 9	IMIT with Plantar flexor contraction;	Mixed	DH ➔ NDH	5	2	400	~100	MIT 10.3 CTR 2.3
Yue and Cole (1992)	7	Male & Female 21–29	MIT = 10 PT = 8 CTR = 9	Abduction of little finger of the hand	Mixed	DH ➔ NDH	4	5	300	~200	MIT 10 PT 14 CTR 2.3
Dahm et al. (2022)	7	Male & Female 18–42	MIT = 21 PT = 21 CTR = 21	EMIT with sequential aiming task	Mirror & Normal	DH ➔ NDH & NDH ➔ DH	1	1	60	~25	DH- > NDH: Mir: MIT 20 PT 25 CTR 12 Nor: MIT 23 PT 23 CTR 17 NDH- > DH: Mir: MIT 21 PT 23 CTR 14 Nor: MIT 26 PT 30 CTR 17
Land et al. (2016)	7	Male & Female 18–24	MIT = 15 PT = 15 CTR = 15	EMIT with sequential tapping task	Mirror & Normal	DH ➔ NDH & NDH ➔ DH	1	1	60	~30	DH- > NDH: Mir: MIT 15 PT 9.5 CTR NC Nor: MIT 19 PT 19 CTR NC NDH- > DH: Mir: MIT 14 PT 10 CTR NC Nor: MIT 17 PT 17 CTR NC
Lohse et al. (2010)	7	NR NR (college students)	MIT = 12 PT = 12	EMIT with writing letter “A”	Normal	DH ➔ NDH & NDH ➔ DH	1	1	8	~10	DH - > NDH: MIT 15 PT 18 NDH - > DH: MIT 27 PT 10
Taktek et al. (2008)	7	Male & Female 8–10	EMIT = 16 IMIT = 16 CTR = 16	EMIT and IMIT with throwing task	Mixed	NDH ➔ DH	1	1	NR	NR	EMIT: MIT NC CTR NC IMIT: MIT 17 CTR NC

SD, standard deviation; MIT, motor imagery training; PT, physical training; CTR, control group; IMIT, internal MIT; EMIT, external MIT; TFR Type, transfer type; TFR DIR, transfer direction; WKs of MIT, weeks of motor imagery training; SESS, sessions; NR, not reported; NC, no change or negative change.

### Reporting bias

**Figure 2:**
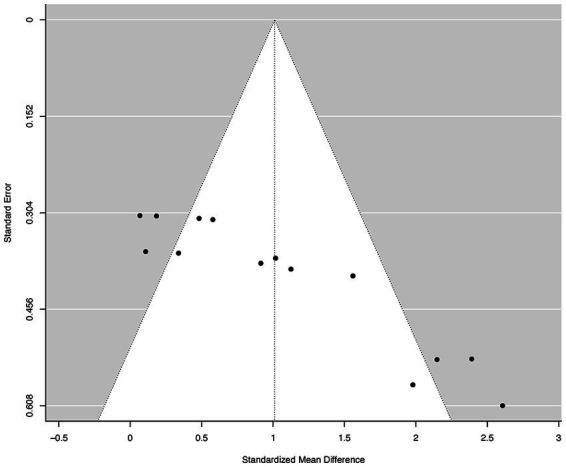
Funnel plots of the standard differences in means vs. standard errors. The aggregated standard difference in means is the random effects mean effect size weighted by the degrees of freedom study characteristics.

Figure 2 displays the funnel plot, indicating possible asymmetry among the analyzed studies. The results of the Egger’s regression test indicated a substantial publication bias, with a value of *p* less than 0.0001.

Following the online database literature search, we identified nine eligible articles reporting on 10 MIT groups (comprising 137 participants) that met our inclusion criteria (refer to Table 1). Table 1 provides an overview of the included articles, outlining information on the sample characteristics, tasks employed, and key findings. Across the selected studies, the sample sizes of the MIT groups ranged from 9 to 21 participants. All of the included studies featured a non-exercise (CTR) and/or a PT.

Five selected studies included both PT and CTR groups (Yue and Cole, 1992; Amemiya et al., 2010; Asa et al., 2014; Land et al., 2016; Dahm et al., 2022). Two studies examined the effect of MIT on BT with children between 8 and 10 years old (Taktek et al., 2008; Asa et al., 2014), while all others had subjects aged 18 to 42 years. Most studies investigated either the effects of internal MIT (IMIT) or external MIT (EMIT), but one study examined both IMIT and EMIT (Taktek et al., 2008). Only three selected studies had multiple training sessions (Yue and Cole, 1992; Alenezi, 2018; Bouguetoch et al., 2021), while all others had only one training session. In addition, the selected studies also varied in testing transfer directions: four studies tested DL ➔ NDL only (Yue and Cole, 1992; Asa et al., 2014; Alenezi, 2018; Bouguetoch et al., 2021), two studies tested NDL ➔ DL only (Taktek et al., 2008; Amemiya et al., 2010), and three studies tested the transfer in both directions (Lohse et al., 2010; Land et al., 2016; Dahm et al., 2022). Furthermore, transfer was investigated in different ways (i.e., mirror, normal, and mixed) across the selected studies. One study used a mirror task only (Amemiya et al., 2010), one study used a normal task only (Lohse et al., 2010), four studies used a mixed task (Yue and Cole, 1992; Asa et al., 2014; Alenezi, 2018; Bouguetoch et al., 2021), and two studies used both mirror and normal tasks (Land et al., 2016; Dahm et al., 2022).

#### Effects of MIT on bilateral transfer (BT) of motor performance

##### Overall effect sizes

**Figure 3:**
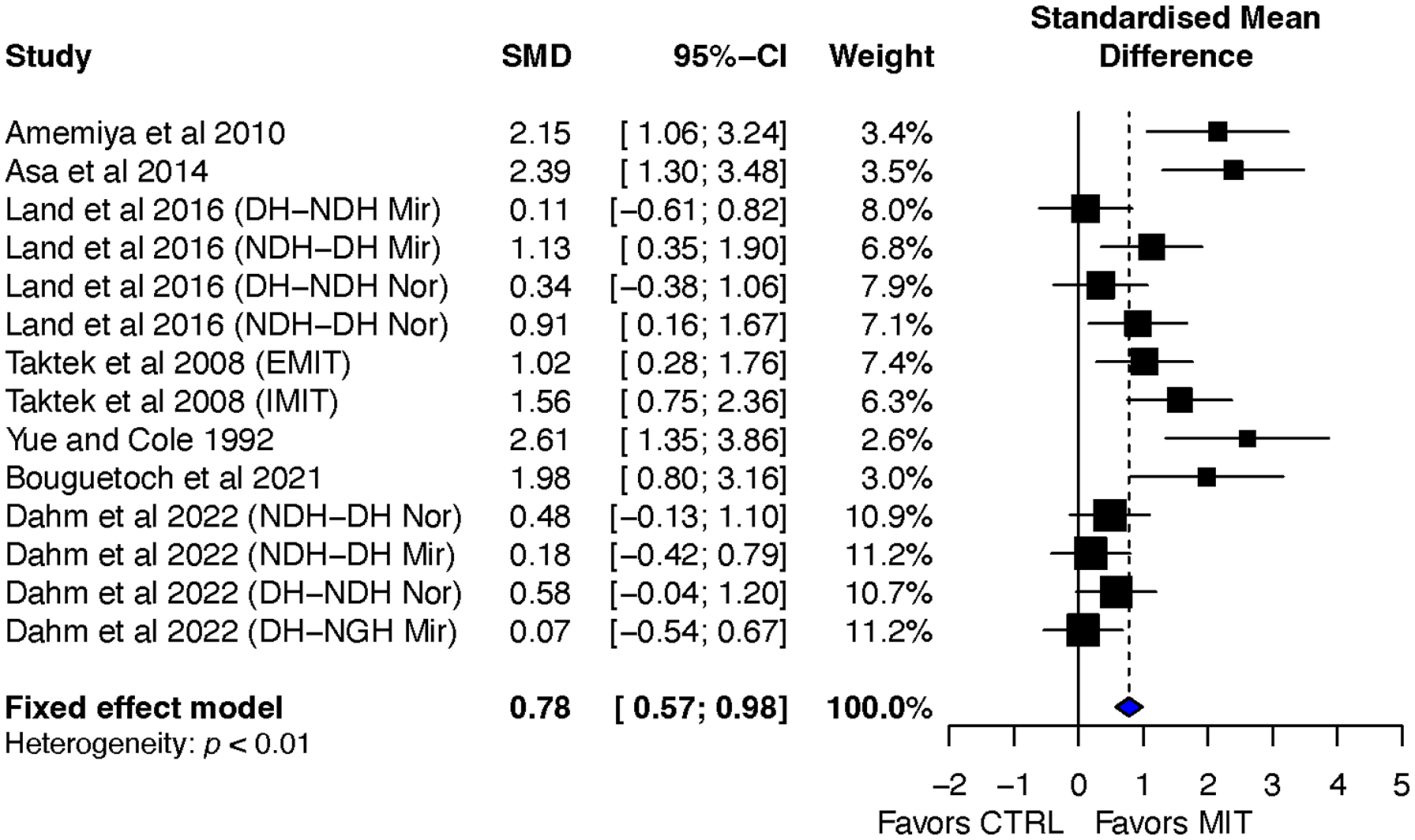
Effects on bilateral transfer: MIT vs. CTR.

In comparison to the CTR, the effect size (ES) of MIT on BT was better for improving the BT effect (ES = 0.78, 95% CI = 0.57–0.98). A quantitative analysis showed a significant between-group difference in the ES, *p* < 0.0001. However, the qualitative overall analysis of all included studies indicated between-study heterogeneity (refer to Figure 3).

A significant result was obtained with the chi-square test for heterogeneity, revealing *p* < 0.01. The I^2^ statistic, calculated to measure the proportion of total variation due to heterogeneity, was found to be 72.1%, indicating medium heterogeneity among the included studies (Higgins et al., 2003).

##### MIT-type subgroup effect sizes

**Figure 4:**
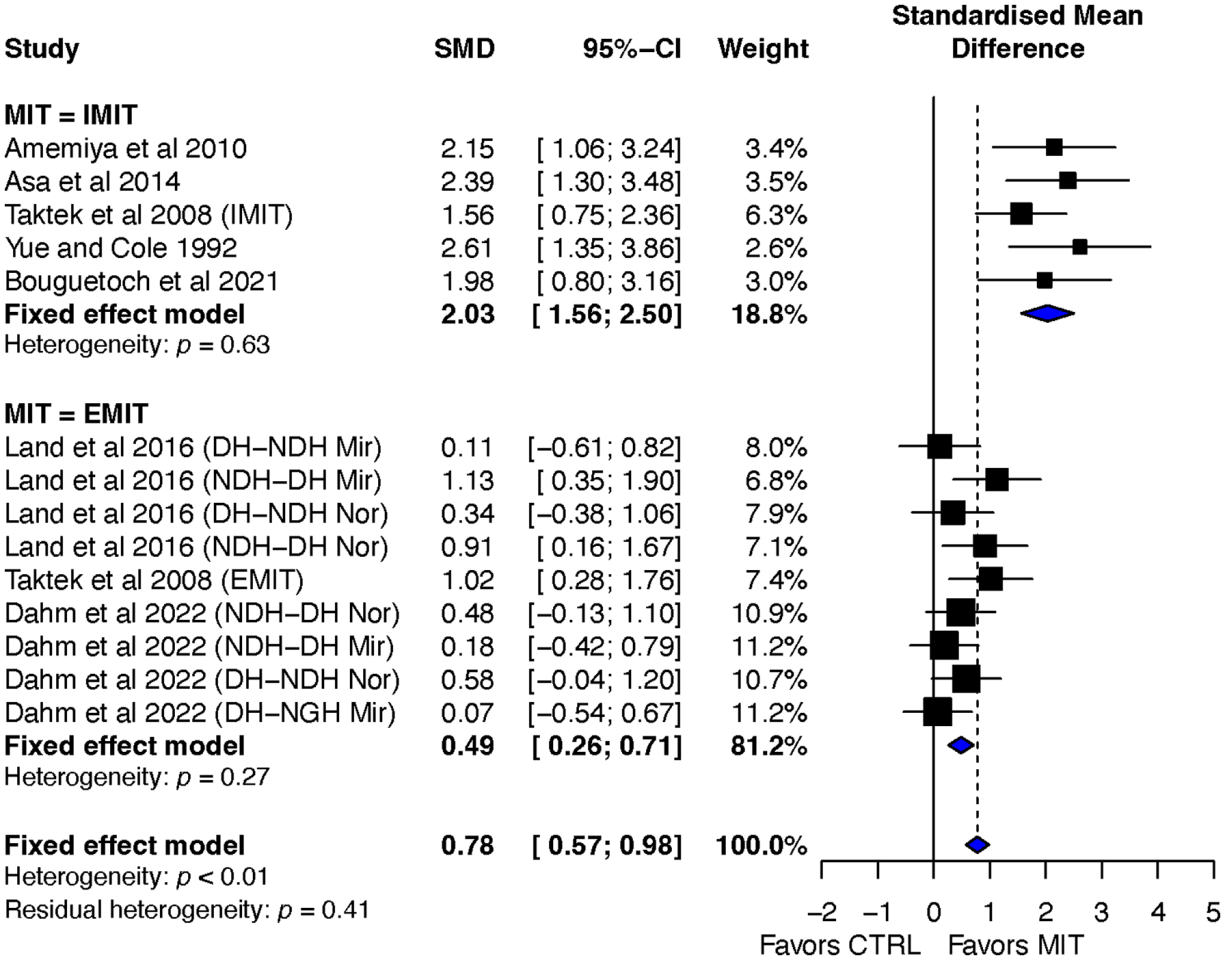
MIT-type subgroup’s effects on bilateral transfer: MIT vs. CTR.

The ES of MIT for inducing BT was found to be more favorable for the IMIT group compared to the EMIT group (ES = 2.03, 95% CI = 1.56–2.50, and ES = 0.49, 95% CI = 0.26–0.71, respectively). The difference between the two groups was significant, Q_1,12_ = 34.08, *p* < 0.0001. The heterogeneity tests for both IMIT and EMIT were not significant, with *p*-values of 0.63 and 0.27, respectively (refer to Figure 4).

##### Transfer-type subgroup effect sizes

**Figure 5:**
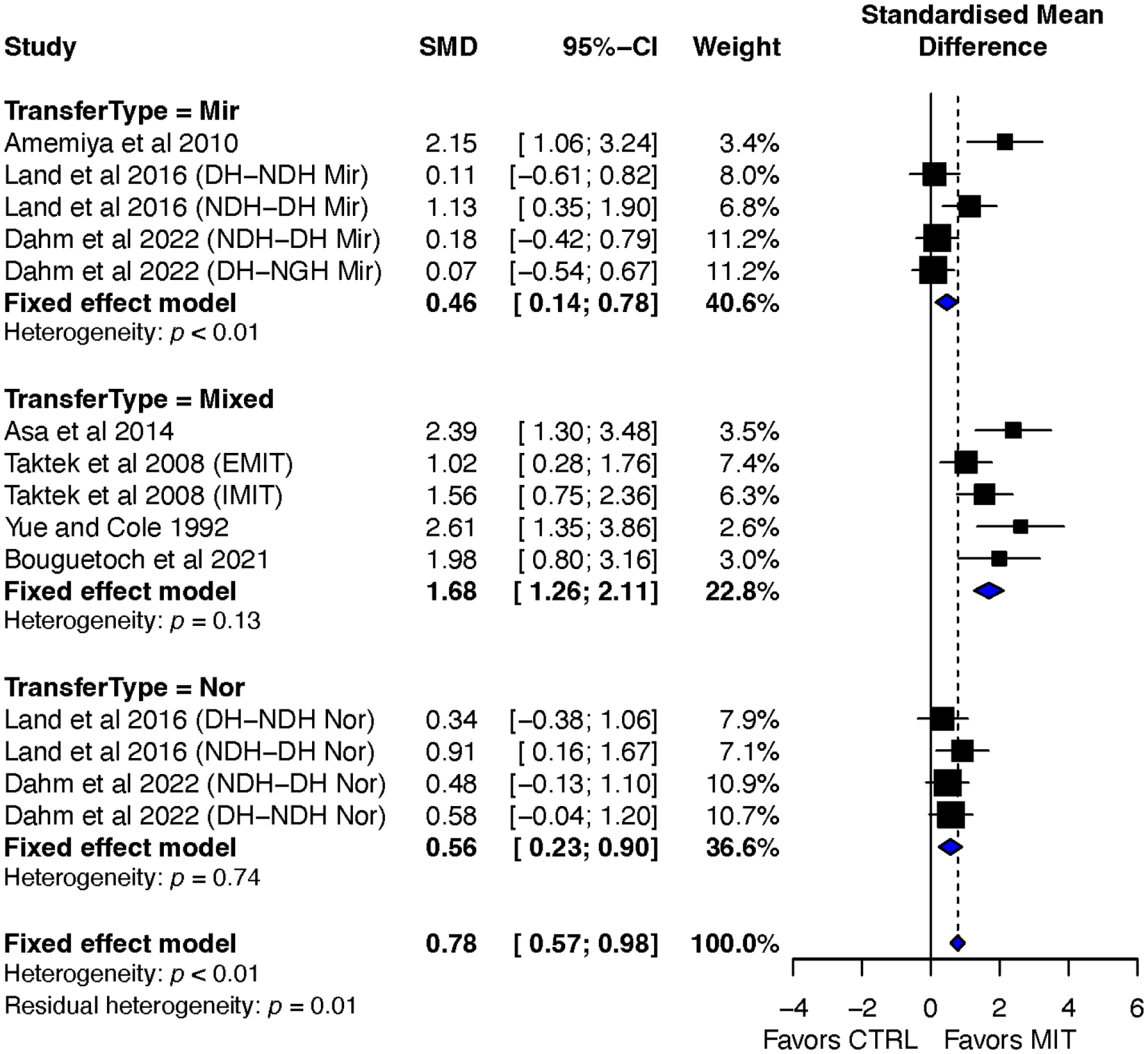
Transfer-type subgroup’s effects on bilateral transfer: MIT vs. CTR.

The ESs of BT for Mixed, Mirror, and Normal orientations were as follows: ES = 1.68, 95% CI = 1.26–2.11; ES = 0.46, 95% CI = 0.14–0.78; and ES = 0.56, 95% CI = 0.23–0.90. The difference between the groups was significant, with Q_2,12_ = 6.39, *p* = 0.012 (refer to Figure 5).

Furthermore, additional quantitative analyses of two-group comparisons on the effects of BT indicated significant differences between the Mixed orientation group and the other two orientation groups. Specifically, Q_1,8_ = 20.38, *p* < 0.0001 for the comparison between the Mixed and Mirror groups, and Q_1,7_ = 16.45, *p* < 0.0001 for the comparison between the Mixed and Normal groups. No significant difference was found between the comparison of Mirror and Normal groups, Q_1,7_ = 0.19, *p* = 0.65. The heterogeneity test was significant for the Mirror group, with p < 0.01, I^2^ = 73.9%. However, for the Mixed and Normal groups, the test was not significant, with *p* = 0.13, I^2^ = 43.8%, and *p* = 0.74, I^2^ = 0.0%, respectfully.

##### Transfer-direction subgroup effect sizes

**Figure 6:**
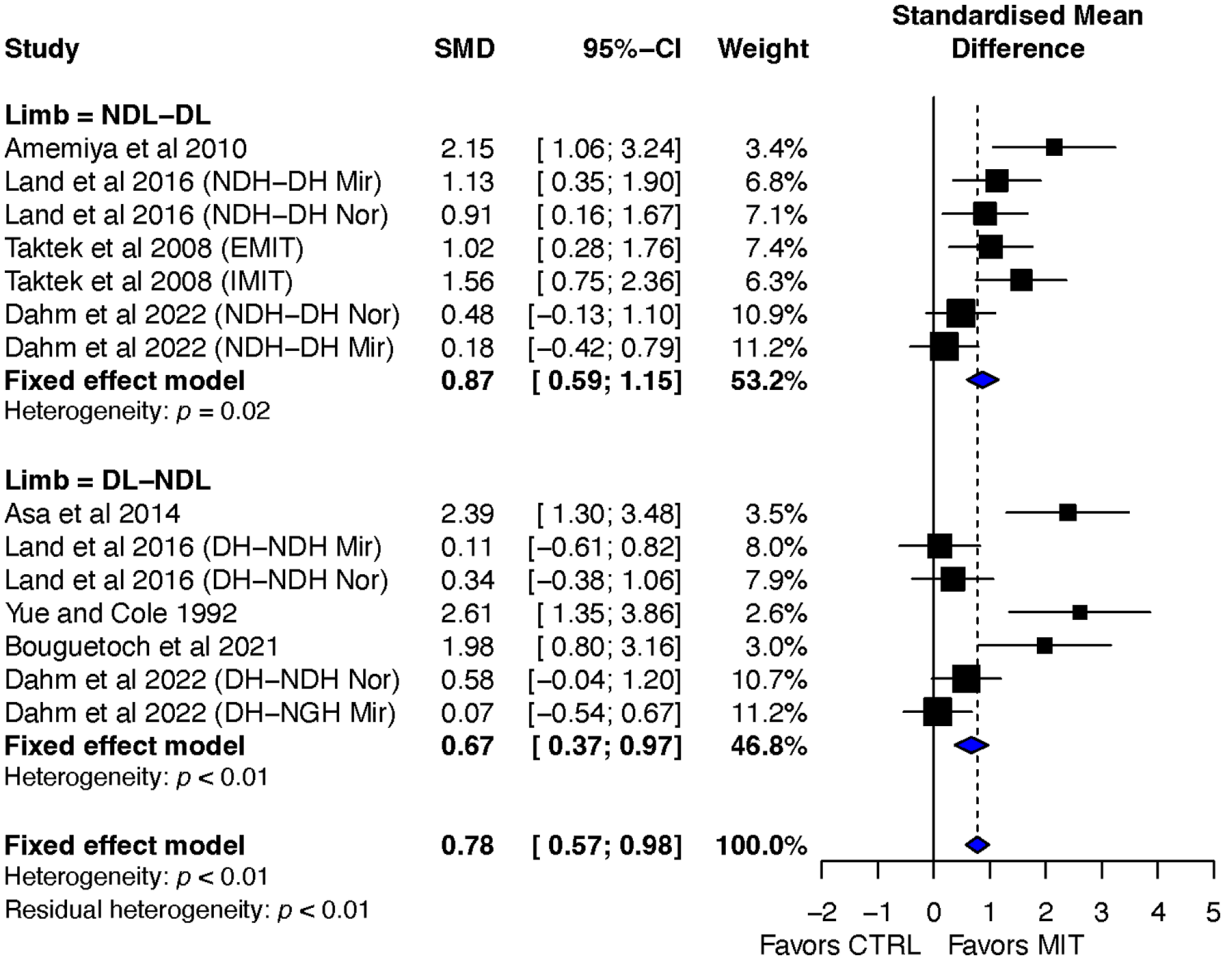
Transfer-direction subgroup’s effects on bilateral transfer: MIT vs. CTR.

Although the ES of BT for NDL to DL was more beneficial than DL to NDL group, ES = 0.87, 95% CI = 0.59–1.15, and ES = 0.67, 95% CI = 0.37–0.97, respectively, the comparison of the two groups was not significant, Q_1, 12_ = 0.94, *p* = 0.33 (Figure 6).

The heterogeneity tests were significant, *p* = 0.02, I^2^ = 15.13% and p < 0.01, I^2^ = 80.3% for NDL to DL and DL to NDL groups, respectfully.

#### MIT effects on BT compared with PT

**Figure 7:**
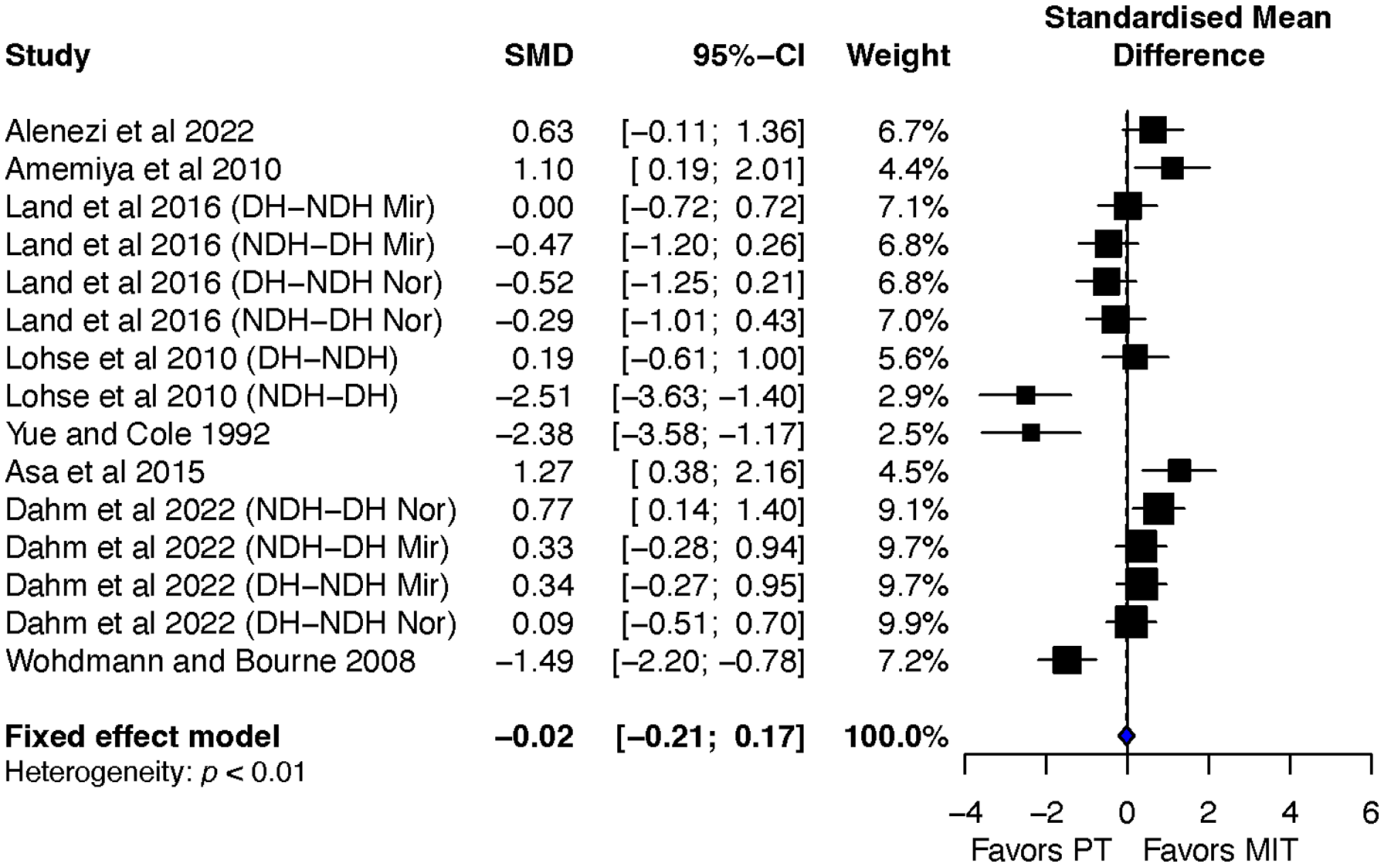
Effects on bilateral transfer: MIT vs. PT.

The ES of MIT in producing a beneficial BT effect was almost identical to the estimated effect of PT (ES = -0.02, 95% CI = -0.21–0.17) (Figure 7). The heterogeneity test was significant, *p* < 0.01, and I^2^ = 82%, indicating substantial heterogeneity among the included studies.

## Discussions

Overall, the aim of this review was to investigate the impact of motor imagery training (MIT) on bilateral transfer (BT) in healthy individuals. The study conducted a systematic review and meta-analysis of randomized controlled studies and found that MIT led moderate but significant improvements in BT when compared to the no-exercise control group (CTR) (see Figure 3), and had a comparable BT effect as physical training (PT) (see Figure 7). The current review also explored the impact of transfer-direction (DL ➔ NDL vs. NDL ➔ DL), transfer-type (mirror vs. normal vs. mixed tasks) and MIT-type (EMIT vs. IMIT) on BT.

### Overall effects of MIT on BT

Previous reviews have indicated a moderate effect of MIT on improving motor skills (Feltz and Landers, 1983; Driskell et al., 1994; Toth et al., 2020) and of MVC force (Paravlic et al., 2018; Meier, 2021; Liu et al., 2023) in the trained limbs. The current review extends this finding to show a moderate overall ES of MIT (ES = 0.78, 95% CI = 0.57–0.98) on BT compared to CTR (Figure 3). However, both qualitative (see Figure 3) and quantitative (Q_13_ = 46.57, *p* < 0.0001, I^2^ = 72.1%) indicated the presence of study heterogeneity among the studies. As argued by Liu et al. (2023), the observed heterogeneity among the included studies could potentially be attributed to differences in the quality of MIT management across the studies. In addition, the differences in MIT-types (i.e., IMIT and EMIT), BT-types (mirror transfer, normal transfer, and mixed transfer) and BT-directions (DL ➔ NDL vs. NDL ➔ DL) might also contribute to the observed heterogeneity.

The beneficial effect of MIT on BT compared to CTR is expected, given that studies (Yue and Cole, 1992; Yao et al., 2013) have shown neural adaptations as a result of MIT similar to those seen with PT. As aforementioned, studies (Imamizu and Shimojo, 1995; Carroll et al., 2006; Lee et al., 2010; Ruddy et al., 2017; Calvert and Carson, 2022) have consistently shown that PT can lead to improvements of the performance in the untrained contralateral limb, known as BT effect. The neural adaptations obtained during PT with the trained limb are believed to contribute to this effect. Therefore, it is reasonable to expect a beneficial effect of MIT on BT.

The current review also reveals a non-significant difference between MIT and PT in leading to BT (ES = −0.02, 95% CI = -0.21–0.17) (Figure 7). This finding may seem unreasonable at first glance since previous studies consistently demonstrate PT’s superiority over MIT for motor skill learning (Feltz and Landers, 1983; Driskell et al., 1994; Toth et al., 2020) or for MVC force enhancement (Paravlic et al., 2018; Meier, 2021; Liu et al., 2023). However, it is important to note that the current review specifically focused on BT resulting from MIT or the MIT effect on the untrained limbs. The bilateral-access hypothesis (Lee and Carroll, 2007) suggests that the neural adaptations induced by PT can be utilized by both the trained and untrained limbs, and consequently resulting in BT effect. Such bilateral accessible neural adaptations had also been reported with MIT that resulted in comparable performance between the trained and untrained limbs (Kohl and Roenker, 1980; Amemiya et al., 2010; Land et al., 2016). This indicates that it is possible for MIT leading to beneficial BT effect.

Notwithstanding, while the bilateral-access assumption could explain why MIT lead to BT, it cannot fully account for why MIT and PT are comparable in inducing BT. Other factors beyond the bilateral-access hypothesis may be responsible for the equality. One possible explanation could be associated with the duration of training. It should be noted that most selected studies in the current review had a single session of training and lasted for less than 30 min (Table 1), which may be sufficient to optimizing the effect of MIT on BT but may not be long enough for PT to realize its full potential. Due to the limited number of studies that used multiple sessions of training and longer training durations (see Table 1), a subgroup analysis could not be conducted to confirm the impact of training duration on the equality of MIT and PT in inducing BT. Future studies should consider investigating the impact of training duration on the effectiveness of MIT and PT in inducing BT, which may provide more insight into the optimal training duration required to fully realize the potential of both training forms.

It is worth noting that it would be valuable to investigate how the combination of MIT and PT (MIT&PT) affects the BT effect. Unfortunately, due to the limited number of studies examining the impact of MIT&PT on BT, the current review was unable to incorporate this moderator into the meta-analysis. However, the most recent review on the effect of MIT on muscle strength (Liu et al., 2023) indicates that MIT&PT is more effective than MIT alone but comparable to PT alone in enhancing muscle strength.

### Impact of MIT-type on BT in motor performance

Based on the findings presented in Figure 4, this review showed the clear superiority of IMIT over EMIT, with a greater ES for IMIT compared to EMIT in BT. Specifically, the estimated effect size was ES = 2.03, 95% CI = 1.56–2.50, for IMIT, and ES = 0.49, 95% CI = 0.26–0.71, for EMIT. The difference between the two groups was statistically significant, with Q_1, 12_ = 34.08, *p* < 0.0001.

This finding is in line with previous reviews on the effects of MIT on motor skill acquisition (Feltz and Landers, 1983; Driskell et al., 1994; Toth et al., 2020) and muscle strength improvement (Paravlic et al., 2018; Meier, 2021; Liu et al., 2023). Furthermore, previous experimental studies (Ranganathan et al., 2004; Yao et al., 2013) have showed that IMIT has greater impact than EMIT in enhancing the power of central drive, as evidenced by increased amplitude of motor-related cortical potential derived from event-triggered scalp EEG data, resulting in improved MVC force.

### Impact of transfer-type on BT in motor performance

The current review also aimed to examine MIT effect on BT by using BT-type or orientation as a moderator. This subgroup analysis consisted of three types: mirror-task, normal-task, and mixed-task. The results of the analysis showed that the mixed-task (ES = 1.68, 95% CI = 1.26–2.11) had a significantly higher treatment effect than the mirror-task (ES = 0.46, 95% CI = 0.14–0.78) and normal-task (ES = 0.56, 95% CI = 0.23–0.90) as indicated in Figure 5. It is worth noting that all individual study point estimates of the mixed-task’s treatment effect (black squares) fell on the right side of the line of no effect (solid vertical line), and, while 3 individual study point estimates and/or the lower limits of CI of the mirror-task and normal-task treatment effect fell on the line of no effect. The non-significant heterogeneity (*p* = 0.13) indicates that the superiority of the mixed-task is even more robust.

The result of this subgroup analysis is not surprising because the mirror-task and normal-task can only access one of the two coordinates (i.e., intrinsic and extrinsic coordinates) and have to modify the other (Lange et al., 2004¸ 2006), while the mixed-task can access both. This means that the mirror-task (or normal-task) requires modifications of the extrinsic coordinates (or intrinsic coordinates) from the learned task with the trained limb, which can lead to deduction of transfer performance with the untrained limb (Lange et al., 2004, 2006). In contrast, the mixed-task can recall both intrinsic and extrinsic coordinates and theoretically has no need for modifications, and consequently leads to better transfer performance.

In sum, the finding from the current review confirmed that the mixed-task, having access to both intrinsic and extrinsic coordinates, performed better in transfers with the untrained limb than tasks having access to just one of the two coordinates (i.e., mirror- and normal-task). It is our understanding that, up to date, this study is the first and only one to compare the transfer effects of these tasks. The finding suggests that therapists should consider mixed-tasks as interventional tasks over normal- or mirror-tasks if the goal is to achieve best transfer performance, such as in rehabilitation for stroke survivors.

### Impact of transfer-direction on BT in motor performance

As aforementioned, transfer-direction (DL ➔NDL or NDL ➔DL) has been one of the most intriguing questions regarding the BT effect. The questions raised regarding the transfer-direction are if a greater amount of BT occurs when a person learns a skill with DL or NDL (asymmetric transfer) or is the amount of transfer similar when either limb is used first (symmetric transfer). The subgroup analysis on transfer-direction found no statistically significant difference between the two groups, Q _1, 12_ = 0.94, *p* = 0.33 (ES = 0.87, 95% CI = 0.59–1.15 for NDL to DL, and ES = 0.67, 95% CI = 0.37–0.97 DL to NDL) as indicated in Figure 6. This finding appears to support the symmetrical transfer hypothesis. However, this assumption is premature since the heterogeneities for the NDL to DL and DL to NDL are significant. The heterogeneity observed in the two groups might be due to the quality of MIT managements across the studies and differences in the characteristics of the tasks in the selected studies. Sainburg and Wang (Sainburg and Wang, 2002; Wang and Sainburg, 2004) have proposed that BT is more asymmetric and its asymmetricity may be linked to the use of different cognitive strategies for different tasks. In other words, while the control center for each limb has access to the information learned during the training of the opposite limb, each control center utilizes this information differently depending on its particular ability to control specific movement features (Sainburg and Wang, 2002). The two studies (Sainburg and Wang, 2002; Wang and Sainburg, 2004) also suggested that visuomotor skills, such as sequential typing tasks, tended to promote transfer from the NDL to the DL, whereas dynamical skills, such as those involved in resistance strength training, tended to promote transfer from the DL to the NDL. The non-significant results between the two transfer directions found in the current review might be due to the limited number of the selected studies testing both transfer directions, which could have influenced the heterogeneity and significance of the results. It should be noted that there were only two studies (Land et al., 2016; Dahm et al., 2022) in the seven selected ones that tested the transfer in both directions. In other words, all the others tested the transfer either from DL to NDL only or NDL to DL only. In sum, the current review’s subgroup analysis on transfer-direction found no statistically significant difference between the two groups, indicating a symmetric transfer. However, due to the limited number of studies that directly tested transfer in both directions, the results cannot draw any overall conclusion on transfer-direction. Therefore, more research is needed to confirm the findings and draw a more definitive conclusion on transfer-direction as a result of MIT.

### Strengths and limitations of the current review

Meta-analysis is an “analysis of analyses” (Glass, 1976), which is a valuable tool for minimizing bias when reviewing evidence from individual studies, as it uses standardized outcome measures and statistical methods to produce a more comprehensive evaluation of the efficacy of interventions like MIT on BT in the present study (Glass, 1976; Borenstein et al., 2009). By employing these methods, this study provides evidence-based answers to important questions regarding the effectiveness of MIT for enhancing BT, as well as the impact of transfer-direction, transfer-type (mirror vs. normal vs. mixed tasks), and MIT-type (EMIT vs. IMIT) on BT.

However, like many other studies with similar designs, the current systematic review and meta-analysis has some limitations that should be considered. For instance, the funnel plot (Figure 2) displays an asymmetric skew, which suggests publication bias. This bias can have implications for the generalization of the findings and may result in an overestimation of the effectiveness of the intervention being studied, in this case, the impact of MIT on BT. This bias could be caused by the fact that studies with statistically significant findings are often more likely to be published than those with non-significant findings. This can lead to an overrepresentation of positive results in the published literature, which can ultimately bias meta-analyses and systematic reviews (Samawi, 2021). The publication bias can also be exacerbated by other factors, such as language restrictions (e.g., only articles published in English selected) and selective reporting of outcomes.

The lack of a sufficient number of studies to allow for subgroup analyses is another limitation that should be addressed in future research. Subgroup analyses can help to identify whether the effectiveness of MIT on BT varies across different subgroups of the population, such as age or task-complexity. This information can be valuable for tailoring interventions to specific subgroups and maximizing their effectiveness. To address this limitation, future studies could focus on recruiting participants from diverse age groups and using tasks with varying levels of cognitive complexity. In sum, future research should consider addressing these limitations to further advance our understanding of the effectiveness of MIT on BT effect.

## Conclusion

In general, the current review shows that MIT generates moderate but significant improvements in BT when compared to the control group (CTR) (Figure 3). While the current review could not draw a definitive conclusion on transfer-direction, it did provide evidence to suggest that transfer-type (mirror vs. normal vs. mixed tasks) and MIT-type (EMIT vs. IMIT) have significant impacts on BT. Additionally, the review suggests that MIT can serve as a valuable alternative or supplement to PT in facilitating beneficial BT effects. Specifically, IMIT is preferable to EMIT, and interventions incorporating tasks that have access to both intrinsic and extrinsic coordinates are preferred over those that involve only one of the two coordinates. This is especially relevant in rehabilitation settings, where patients with motor disabilities, such as stroke survivors, can benefit from such interventions.

## Author contributions

WXY, SG and GHY had substantial contributions to the design of the work and interpretation of the data. All authors contributed equally on drafting the manuscript and agree to be accountable for all aspects of the work in ensuring that questions related to the accuracy or integrity of any part of the work are appropriately investigated and resolved.

## Conflict of interest

The authors declare that the research was conducted in the absence of any commercial or financial relationships that could be construed as a potential conflict of interest.

## Publisher’s note

All claims expressed in this article are solely those of the authors and do not necessarily represent those of their affiliated organizations, or those of the publisher, the editors and the reviewers. Any product that may be evaluated in this article, or claim that may be made by its manufacturer, is not guaranteed or endorsed by the publisher.
